# The impact of the COVID-19 pandemic on ST-segment elevation myocardial infarction treatment strategy and outcomes

**DOI:** 10.3389/fcvm.2025.1522661

**Published:** 2025-04-16

**Authors:** Jakub Bychowski, Tomasz Michalski, Wojciech Sobiczewski, Miłosz Jaguszewski, Marcin Gruchała

**Affiliations:** 1st Department of Cardiology, Medical University of Gdansk, Gdansk, Poland

**Keywords:** COVID-19 pandemic, OTDT, STEMI, time from admission to wire crossing, quality of care

## Abstract

**Background:**

The most reliable care quality indicators for STEMI patients undergoing primary percutaneous coronary intervention (pPCI) include onset-to-door time (OTDT), time from admission to wire crossing and in-hospital mortality.

**Aims:**

Our study aimed to evaluate the impact of the COVID-19 pandemic on these selected care quality indicators in pre-pandemic and pandemic groups of STEMI patients.

**Methods:**

This single-centre, retrospective study, enrolled 480 STEMI patients, aged 63.59 ± 12.44 years treated with pPCI across two time frames: pre-pandemic (*n* = 331) and pandemic (*n* = 149). The evaluation criteria included OTDT, time from admission to PCI-mediated reperfusion, in-hospital mortality, and predictors of time delays.

**Results:**

Our study revealed a significant increases in OTDT (median 3 h; IQR 1.5–12.0 vs. median 5 h; IQR 2.0–24.0, *p* = 0.011) and time from admission to wire crossing (median 92 min; IQR 65.0–187.0 vs. median 115.0; IQR 73.0–233.0, *p* = 0.025), in the COVID-19 pandemic group of STEMI patients, compared to the pre-pandemic subset. We also observed an increase in in-hospital mortality (7.85% vs. 14.09%, *p* = 0.033) and incidence of cardiogenic shock/cardiac arrest (16.62% vs. 26.85%, *p* = 0.009). Additionally, the proportion of patients with prolonged OTDT (24.45% vs. 35.71%, *p* = 0.019) and extended time from admission to PCI-mediated reperfusion (51.96% vs. 65.77%, *p* = 0.005) increased during the pandemic period.

**Conclusions:**

The study's results indicated prolonged OTDT and admission-to-wire crossing times, increased in-hospital mortality, and the higher frequency of cardiogenic shock/cardiac arrest during the COVID-19 pandemic. These findings demonstrate the negative impact of the pandemic on treatment times and outcomes for patients diagnosed with STEMI.

## 1 Introduction

The first reports about the outbreak of the Coronavirus Disease 2019 (COVID-19) started at the end of December 2019 and were enhanced by the World Health Organization at the beginning of January 2020. The disease rapidly grew into a pandemic, officially announced on the 11th of March, 2020 (1). As a result, the Polish government established numerous limitations, that were sustained continuously for approximately 3 months, until the 30th of May, 2020.

As a result, the Ministry of Health authorized the system of unitary hospitals dedicated only to treating COVID-19 individuals, in contrast to multi-specialist centres designed for patients with other diseases. In these hospitals scheduled admissions were restrained, with exception for oncological, obstetric and paediatric.

The fundamental care quality indicators for ST-segment elevation myocardial infarction (STEMI) treatment remained unchanged. Following the European Society of Cardiology (ESC), adequate indicators of care quality are associated with treatment delay, as the patient's primary condition is irrelevant in such measures. Moreover, the most capable factor of the treatment outcome remained in-hospital and 30-day mortality (2).

The rapid healthcare system reorganisation must influence the quality of care in patients treated with Acute Coronary Syndrome (ACS), especially those with STEMI. The only available study in Poland revealed that 7.4% of cardiac catheterization centres in Poland were timely closed. The authors reported a relevant decrease in patient admission from all types of ACS, including STEMI, where the deterioration reached 36% when comparing the 2-months observation period (3). Similar alterations were observed in European countries affected by Severe Acute Respiratory Syndrome Coronavirus 2 (SARS-CoV2). De Rosa et al. revealed a 48.4% decline in admission for ACS, with STEMI reduction at 26.5% over 1 week (4). Another multi-centre, retrospective study including 77 European centres reported an 18.9% decrease in the reported number of STEMI cases, comparing 2019–2020. Notably, a significant increase in in-hospital mortality occurred (6.8% vs. 4.9%, *p* < 0.001) (5). In response to the developing crisis in the healthcare system, in the middle of May 2020, the ESC published a Position Paper on the Invasive Management of ACS (6).

None of the published studies evaluated mortality and treatment delays in patients diagnosed with STEMI in Poland during the COVID-19 pandemic irrespectively of infection status and compared it to the cohort enrolled before the pandemic onset. Considering existing disparities between countries, assessing these rates in our population is indispensable. Thus, the current study aimed to evaluate the influence of the COVID-19 pandemic on STEMI treatment in Poland by analysis of the following indicators: onset-to-door time (OTDT), time from admission to wire crossing, and in-hospital mortality in pre-pandemic and pandemic groups. In addition, disparities in established risk factors and the number of hospitalised cases between the groups were identified.

## 2 Methods

This was a retrospective study including 480 patients (328 males, 68.48%), with a mean 64 ± 12.44 years of age consecutively admitted to the catheterization laboratory at the University Clinical Centre (UCC) in Gdańsk, Poland in three following ways: transported by Emergency Medical Services (EMS) (*n* = 390; 81%), admitted personally to the Emergency Unit (EU) of the UCC (*n* = 70; 15%), or referred from the other ward of UCC (*n* = 20; 4%). All included patients were initially diagnosed with STEMI and treated by pPCI. The diagnosis was based on the ESC guidelines (2). There were two-time frames for including patients in our study: pre-pandemic (from January 1, 2015, to December 31, 2016) and pandemic (from March 22, 2020, to March 30, 2021). Patients with unknown OTDT, time from admission to PCI-mediated reperfusion, or transported to the EU of UCC from peripheral hospitals without a catheterization laboratory were excluded. Cases of cardiac arrest (both out and in-hospital) and cardiogenic shock were included in this study. To eliminate the potential influence of COVID-19 infection on results, we excluded COVID-19-positive patients from the final analysis. Thus, due to the reported decrease in the number of patients admitted to the hospitals during the COVID-19 pandemic, we estimated the number of patients introduced to the UCC with the diagnosis of STEMI in the pre-pandemic and pandemic groups.

The data regarding every incorporated patient were collected: medical history, laboratory test results, transthoracic echocardiography (TTE) parameters, mortality risk assessment, short-term outcome markers, and time frames recommended by ESC as the quality-of-care indicators.

The present study complies with the Declaration of Helsinki. Furthermore, the methods used in this analysis were reviewed and approved by the Institutional Review Board at the UCC (134/2021) and the Local Independent Bioethical Committee (NKKBN/604/2021).

### 2.1 COVID-19 prevention procedure

According to the ESC Position Statement, every patient with unknown COVID-19 status was treated as potentially infected (6). pPCI remained the therapy of choice in cases where it can be potentially delivered in the recommended time limit. SARS-CoV2 testing was performed in all patients referred to the EU. Initially, nasopharyngeal swab sample was taken for antigenic testing and additional swab was taken to conduct polymerase chain reaction (PCR) testing for result confirmation. In urgent cases, a patient can be transported to receive PCI after receiving negative antigenic tests. The patient must be isolated in a designated room until a negative PCR result is obtained. Medical professionals managing a patient with unknown COVID-19 status were supplied with personal protective equipment (PPE), including an N95 respirator, gloves, and surgical cap.

### 2.2 Medical history

The following baseline characteristics of each included patient were collected on the day of the admission: age, sex, weight, height, comorbidities, previous diseases or procedures, administered drugs, the reason for admission, family history and COVID-19 status at admission.

### 2.3 Laboratory testing

The analysed results of laboratory tests, such as haemoglobin, platelets count, and biochemistry analysis (including cardiac biomarkers: hsTnI, CK-MB), were conducted on admission. All laboratory analyses were performed in the Central Clinical Laboratory of UCC.

### 2.4 Echocardiography

TTE was conducted on each patient upon admission. The study reported the following variables: left ventricular ejection fraction (LVEF) and indirect signs of pulmonary hypertension.

### 2.5 Mortality risk assessment

Every enrolled patient was evaluated on the day of admission by the following clinical scales: the Canadian Cardiac Society (CCS) scale, the New York Heart Association (NYHA) scale, and the Killip-Kimball scale. Estimating the six-month mortality risk was conducted using the GRACE 2.0 scale. The peri-procedural risk of death was assessed using the Euro score 2 scale.

### 2.6 Short-term outcome assessment

The treatment outcomes were estimated by reporting in-hospital mortality and the occurrence of cardiac arrest or cardiogenic shock both out of- and in-hospital.

### 2.7 Delays in the pre- and in-hospital setting

In each group, the following care quality indicators were assessed: OTDT—the time from the onset of symptoms to patient admission to the UCC, the time from patient admission to the UCC to wire crossing in the culprit lesion. Data about symptoms onset were collected from direct patient interviews or EMS information cards. The precise admission and procedure times were assembled from electronic documentation stored in UCC.

### 2.8 Comparison of the pre- and pandemic period

In consideration to the aims of the study, we divided the population into two groups: pre-pandemic (admitted from January 1, 2015, to December 31, 2016, *n* = 331) and pandemic (admitted from March 22, 2020, to March 30, 2021, *n* = 149). To evaluate the influence of the COVID-19 pandemic on the healthcare system in Poland, we subsequently compared collected parameters in both groups. Finally, PCR testing assessed in-hospital mortality in patients positive for SARS-CoV2 (*n* = 4; 2.68%).

### 2.9 Statistical analysis

Descriptive statistics were performed for each cohort of patients, including the baseline characteristics of the analysed population and characteristics of the subgroups, here depending on the specific parameter. Continuous variables are presented as the mean ± standard deviation or median with interquartile range according to the data distribution. Dichotomous variables are presented as counts with percentages. The data's normality and variance homogeneity were determined using Shapiro–Wilk and Levene's tests. Furthermore, the t-test or the Mann–Whitney U test was used to compare the continuous variables. The dichotomous variables were compared using the chi-square test. *P*-values of <0.05 were considered to indicate statistical significance. Statistical analyses were performed using Statistica 13.3 (StatSoft, TIBCO Software Inc.).

## 3 Results

The demographic and baseline characteristics of the analysed population are presented in Table 1. The median OTDT in the study population was 3.5 h (IQR 1.5–15.0), and it was prolonged (>12 h) in 117 cases (27.86%). The median time from admission to wire crossing was 99.5 min (IQR 66.5–199.5), and it was prolonged (≥90 min) in 270 cases (56.25%). The in-hospital mortality rate in the whole study group was 9.79%. Cardiogenic shock was reported in 47 cases (9.81%), cardiac arrest occurred in 82 of patients (17.08%).

**Table 1:** Demographic and baseline characteristics of the study population.

Parameter^a^	Patients (*n* = 480)
Age [years]	63.59 ± 12.44
Length of hospitalization [days]	6.00 (4.00–10.00)
Male [%]	328 (68.33)
Weight [kg]	80.00 (71.00–92.00)
Height [cm]	170.50 (165.00–176.00)
Obesity [%]	141 (31.33)
BMI [kg/m^2^]	27.68 (24.55–31.00)
Hypertension [%]	304 (65.24)
Smoking	137 (30.11)
Renal failure [%]	22 (4.82)
Dyslipidaemia [%]	316 (67.81)
Diabetes mellitus [%]	101 (21.81)
Chronic lung disease [%]	21 (4.53)
Cardiogenic shock [%]	47 (9.81)
Cardiac arrest [%]	82 (17.08)
Out-of-hospital cardiac arrest [%]	49 (10.21)
hsTnI [ng/ml]	28.29 (6.65–68.77)
CK-MB [ng/ml]	51.80 (14.00–132.20)
Family history of CAD [%]	83 (22.37)
Extracardiac arteriopathy [%]	29 (6.29)
LVEF [%]	45.00 (36.00–50.00)
Grace score—in hospital	121.00 (102.00–148.00)
Creatinine level [mg/dl]	0.92 (0.78–1.15)
VKA	11 (2.70)
NOAC	14 (9.40)

Abbreviations: ACEI, angiotensin converting enzyme inhibitors; ARB, angiotensin II receptor blockers; BMI, body mass index; CAD, coronary artery disease; CK-MB, creatine kinase MB; hsTnI, high sensitive troponin I; LVEF, left ventricle ejection fraction; VKA, vitamin K antagonists.

^a^ Data are presented as mean ± standard deviation, median (interquartile range), or counts (percentages).

### 3.1 Pre-pandemic period

A detailed analysis of the group was conducted in our previous study (7).

### 3.2 Pandemic period

As in the pre-pandemic group, we analysed potential risk factors of OTDT and time from admission to PCI-mediated reperfusion prolongation. Results are summarised in Table 2. The median of OTDT was 5.0 h (IQR 2.0–24.0) and prolonged in 45 cases (35.71%). The following factors were related to OTDT prolongation: lower height, the incidence of dyslipidaemia, longer time from admission to wire crossing, lower CCS score, lower hsTnI, and CK-MB results. No significant differences were reported for the following variables: age, sex, time of admission, the incidence of obesity, diabetes mellitus (DM), smoking, renal failure, cardiogenic shock, cardiac arrest, out-of-hospital cardiac arrest or administration of any medication. The analysis revealed the median time from admission to PCI-mediated reperfusion was 115.0 min (IQR 73.0–233.0), the prolongation occurred in 98 cases (65.78%). In this group, prolonged time was correlated with longer OTDT, lower incidence of chronic obstructive pulmonary disease, administration of antagonists of vitamin K (VKA) and angiotensin receptor blockers (ARB), higher administration of ACE inhibitors (ACEI) and incidence of extracardiac arteriopathy. No differences between groups existed for age, sex, time of admission, length of hospitalisation, the incidence of obesity, dyslipidaemia, DM, smoking, renal failure, type of admission, cardiogenic shock, cardiac arrest, out-of-hospital cardiac arrest. Moreover, prolonged time from admission to wire crossing in patients from the pandemic group was related to a higher rate of in-hospital mortality than pre-pandemic (18.37% vs. 5.88%; *p* = 0.038). Consistently to our previous observations, admission during the nightshift did not affect in-hospital mortality (17.65% vs. 13.95%; *p* = 0.65).

**Table 2:** Predictors of OTDT and time from admission to wire crossing prolongations in pandemic group.

Parameter^a^	Onset-to-door-time			Time from admission to wire crossing		
	≤12 h (*n* = 81)	>12 h (*n* = 45)	*P*-value	<90 min (*n* = 51)	≥90 min (*n* = 98)	*P*-value
Age [years]	65.00 (58.00–73.00)	66.00 (59.00–73.00)	0.518	65.00 (57.00–73.00)	68.00 (59.00–74.00)	0.333
Females	26/81 (32.10)	20/45 (44.44)	0.169	19/51 (37.25)	36/98 (36.73)	0.950
Weight [kg]	81.56 ± 14.97	80.49 ± 16.42	0.721	80.48 ± 15.57	81.48 ± 15.73	0.724
Height [cm]	171.36 ± 8.13	168.29 ± 7.77	**0**.**0496**	170.92 ± 8.44	169.87 ± 8.11	0.482
Hypertension	54/81 (66.67)	24/45 (53.33)	0.140	31/51 (60.78)	59/94 (62.77)	0.814
Renal failure	0/81 (0.0)	1/45 (2.22)	0.178	0/51 (0.0)	4/96 (4.17)	0.139
Dyslipidaemia	55/81 (67.90)	21/45 (46.67)	**0**.**020**	32/51 (62.75)	54/96 (56.25)	0.447
Diabetes on insulin	4/81 (4.94)	3/45 (6.67)	0.685	5/51 (9.80)	4/97 (4.12)	0.169
Chronic lung disease	3/81 (3.70)	0/45 (0.0)	0.838	6/51 (11.76)	1/96 (1.04)	**0**.**004**
Cardiogenic shock	9/81 (11.11)	4/45 (8.88)	0.694	6/51 (11.76)	18/98 (18.37)	0.298
Cardiac arrest	16/81 (19.75)	6/45 (13.33)	0.363	9/51 (17.65)	24/98 (24.49)	0.34
Out-of-hospital CA	7/81 (8.64)	1/45 (2.22)	0.157	6/51 (11.76)	11/98 (11.22)	0.922
hsTnI [ng/ml]	47.79 (15.23–85.50)	21.32 (8.35–51.69)	**0**.**016**	37.32 (13.23–103.73)	25.31 (8.35–60.17)	0.089
CK-MB [ng/ml]	82.30 (23.90–173.20)	29.00 (8.50–91.80)	**0**.**003**	65.75 (19.50–143.80)	47.85 (15.30–141.80)	0.612
CCS IV	56/71 (78.87%)	21/40 (52.5%)	**0.004**	32/42 (76.19%)	47/77 (61.04%)	0.09
In-hospital mortality	7/81 (8.64)	6/45 (13.33)	0.407	3/51 (5.88)	18/98 (18.37)	**0**.**038**
VKA	1/75 (1.33)	0/43 (0.0)	0.447	2/47 (4.26)	0/90 (0.0)	**0**.**049**
ACEI	18/75 (24.00)	9/43 (20.93)	0.702	6/47 (12.77)	28/90 (31.11)	**0**.**018**

Abbreviations: ACEI, angiotensin converting enzyme inhibitors; CA, cardiac arrest; CCS, Canadian Cardiovascular Society; CK-MB, creatine kinase MB; CS, cardiogenic shock; hsTnI, high sensitive troponin I; VKA, vitamin K antagonists.

Bolded values present statistically significant results of the analysis.

^a^ Data are presented as mean ± standard deviation, median (interquartile range), or counts (percentages).

### 3.3 Percutaneous coronary intervention

Details of PCI and its complications are presented in Tables 3, 4. Patients from pandemic group had more severe CAD and received trombectomy more often, compared to pre-pandemic group. In the pandemic period no reflow and minor bleeding occurred more often. Cardiac arrest during the procedure was more often reported in pre-pandemic group.

**Table 3:** Percutaneous coronary intervention details.

Parameter^a^	Pre-pandemic group	Pandemic group	*P*-value
Radial approach during PCI	294/324 (90.74)	135/149 (90.6)	0.962
Number of stenosis			
1	166/323 (51.39)	36/149 (24.16)	**<0**.**001**
2	83/323 (25.7)	50/149 (33.56)	
3	51/323 (15.79)	43/149 (28.86)	
4	23/323 (7.12)	20/149 (13.42)	
*de novo* stenosis	306/323 (94.74)	145/149 (97.32)	0.207
Aspiration thrombectomy during PCI	99/323 (30.65)	62/149 (41.61)	**0**.**02**
2nd generation DES implantation	258/312 (82.69)	129/148 (87.16)	0.22
Incidence of residual stenosis	25/310 (8.06)	14/146 (9.59)	0.587
TIMI 3	288/313 (92.01)	136/146 (93.15)	0.669

Abbreviations: DES, drug eluting stent; PCI, percutaneous coronary intervention; TIMI, thrombolysis in myocardial infarction risk score.

Bolded values present statistically significant results of the analysis.

^a^ Data are presented as mean ± standard deviation, median (interquartile range), or counts (percentages).

**Table 4:** Perirocedural complications details.

Parameter^a^	Pre-pandemic group	Pandemic group	*P*-value
Periprocedural MI	2/312 (0.64)	1/149 (0.67)	0.97
Perforation	0/311 (0)	0/149 (0)	1.00
Dissection	6/312 (1.92)	3/149 (2.01)	0.948
No reflow	5/312 (1.6)	14/149 (9.4)	**<0**.**001**
Cardiac arrest	35/331 (10.57)	6/149 (4.03)	**0**.**018**
IABP implantation	11/331 (3.32)	8/149 (5.37)	0.288
Bleeding without transfusion	1/331 (0.3)	12/149 (8.05)	**<0**.**001**
Bleeding with transfusion	2/331 (0.6)	1/149 (0.67)	0.931
Acute stent thrombosis	2/330 (0.61)	1/149 (0.67)	0.933

Abbreviations: IABP, intra-aortic balloon pump; MI, myocardial infarction.

Bolded values present statistically significant results of the analysis.

^a^ Data are presented as mean ± standard deviation, median (interquartile range), or counts (percentages).

### 3.4 Pre- vs. pandemic period

**Figure 1:**
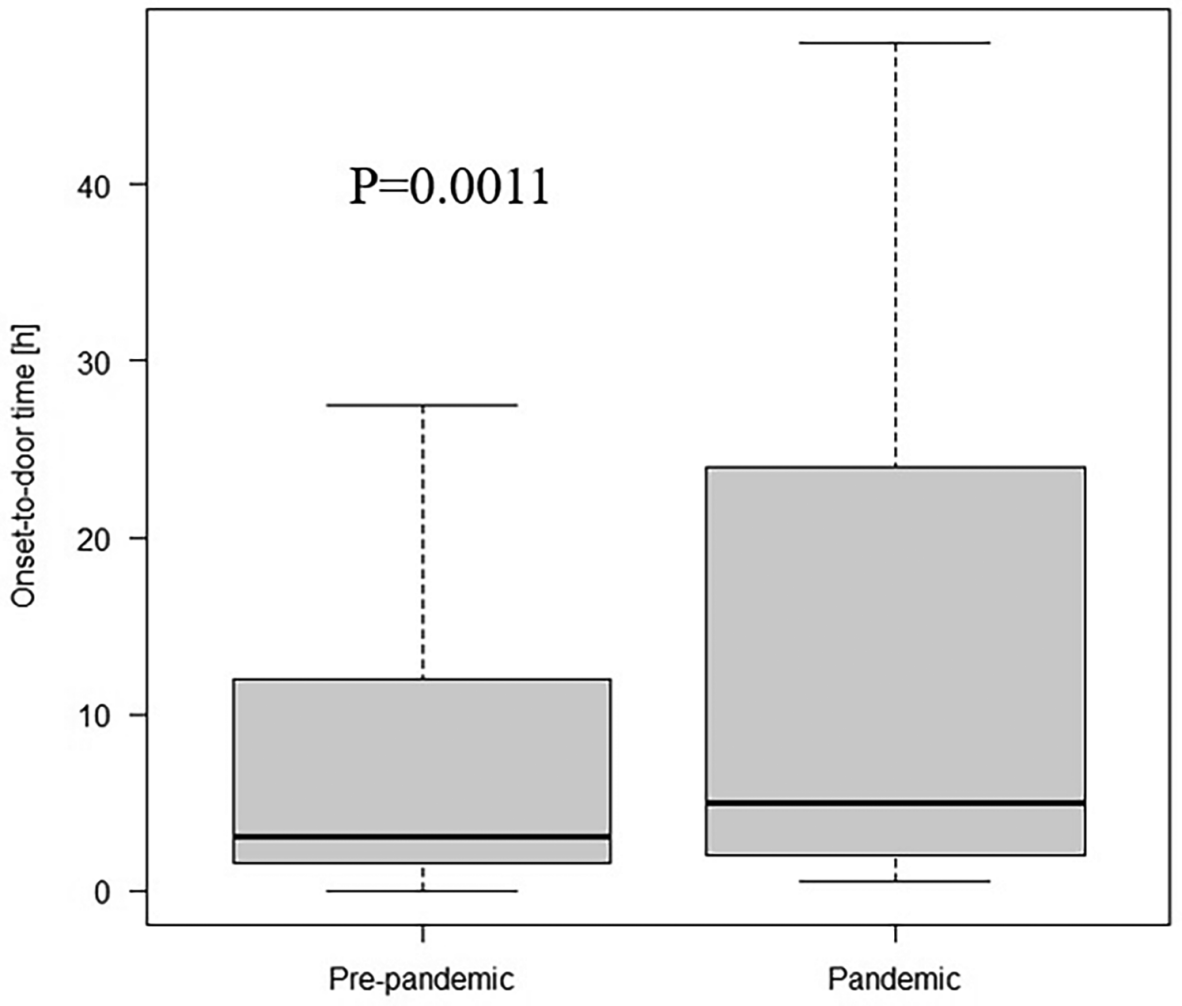
Onset-to-door time in the study cohort divided into pre-pandemic and pandemic group.

**Figure 2:**
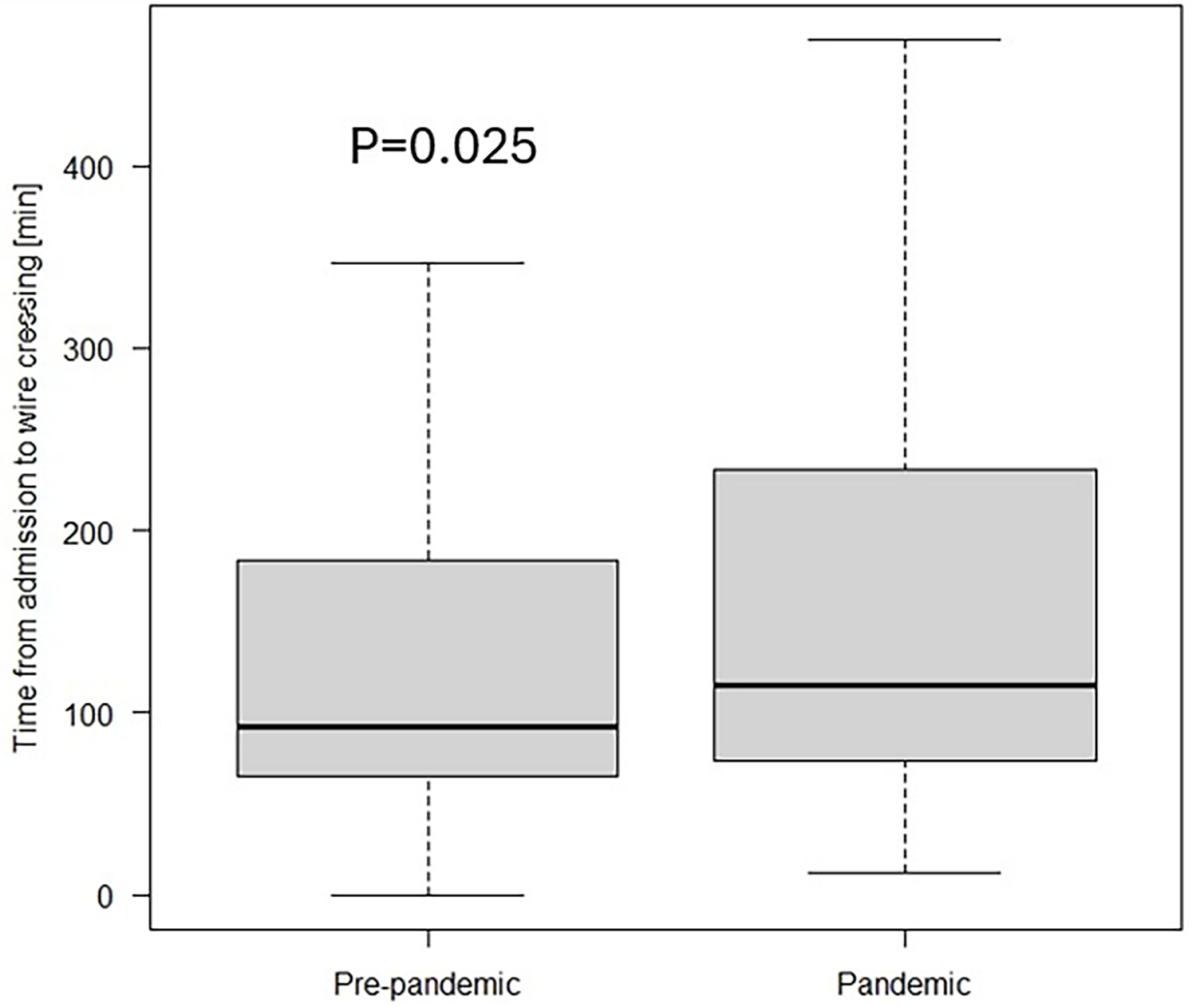
Time from admission to wire crossing in the study cohort divided into pre-pandemic and pandemic group.

**Figure 3:**
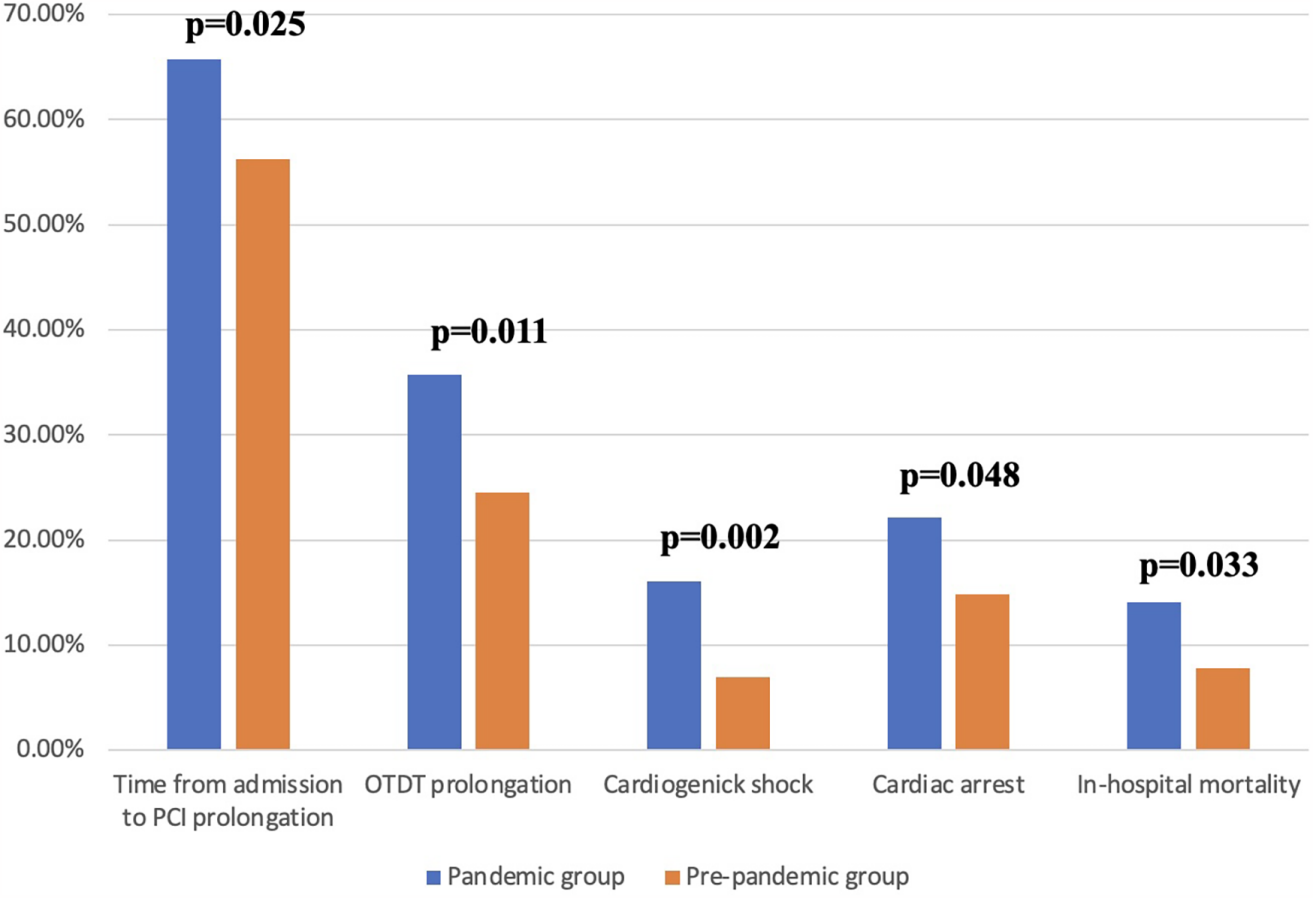
Percentage of indicators occurence in the study groups. OTDT, onset-to-door time; PCI, percutaneous coronary intervention.

The comparison between the groups is presented in Table 5. When divided into pre-pandemic and pandemic groups, patients in the pandemic group had a greater prevalence of administration of calcium channel blocker (CCB) and ARB at admission and higher scores in the NYHA, Killip-Kimball, and GRACE 2.0 scale. Further, we observed a lower frequency of dyslipidaemia, smoking history, and acute myocardial infarction (AMI) in the pandemic group, a lower score in the CCS and Euro score 2 scales. There were no significant differences in age, sex, type of clinical presentation, or LVEF measured by TTE on the admission day. Moreover, reduced hospitalisation length was noted in the pandemic group, although this disparity did not achieve a statistical significance (6.0 vs. 5.0; *p* = 0.075). After partitioning into groups, the median of OTDT was longer in the pandemic group (3.0 h; IQR 1.5–12.0 vs. 5.0 h; IQR 2.0–24.0, *p* = 0.011, Figure 1). Prolonged OTDT was observed more frequently in the pandemic group (24.49% vs. 35.71%, *p* = 0.019). Similarly, the median time from admission to wire crossing was significantly higher in the pandemic group than in the pre-pandemic group (92.0 min; IQR 65.0–187.0 vs. 115.0 min; IQR 73.0–233.0, *p* = 0.025, Figure 2). The prolongation occurred more often in the pandemic group (51.96% vs. 65.78%, *p* = 0.005). Moreover, the incidence rates of cardiogenic shock (6.97% vs. 16.11%, *p* = 0.002) and cardiac arrest (14.8% vs. 22.15%, *p* = 0.048) were more prevalent in the pandemic group, as well as the mortality rate (7.85% vs. 14.09%, *p* = 0.033). All disparities between those variables are presented on Figure 3. After excluding SARS-CoV2 positive patients, disparities in mortality (7.85% vs. 13.79%, *p* = 0.044), cardiogenic shock (6.97 vs. 15.17%, *p* = 0.004) and cardiac arrest (14.8% vs. 22.76%, *p* = 0.034) remained significant. We identified 4 patients with COVID-19 infection admitted with STEMI diagnosis. There was one (25%) reported case of death in this group, cardiogenic shock was reported in 2 cases (50%). There was no difference in the distribution of admission time between the group (17.65% of patients from pandemic group were admitted on night shift, compared to 13.95% from pre-pandemic group, *p* = 0.65).

**Table 5:** Comparison between pre-pandemic and pandemic group.

Parameter^a^	Pre-pandemic group	Pandemic group	*P*-value
Age [years]	62.95 ± 12.23	65.03 ± 12.84	0.089
Length of hospitalization [days]	6.0 (4.0–10.00)	5.00 (4.00–9.00)	0.075
Female	96/331 (29.09)	55/149 (36.91)	0.088
Weight [kg]	80.0 (71.0–92.0)	80.0 (70.0–90.0)	0.503
Height [cm]	172.0 (165.0–176.0)	170.0 (165.0–176.0)	0.146
BMI [kg/m^2^]	27.70 (24.54–30.67)	27.0 (25.0–31.0)	0.837
Hypertension	214/320 (66.67)	90/145 (62.07)	0.335
Dyslipidaemia	230/319 (72.10)	86/147 (58.50)	**0**.**0035**
Diabetes mellitus	66/316 (20.88)	35/147 (23.81)	0.442
Smoking history	114/309 (36.89)	26/139 (18.71)	**<0**.**001**
Cardiogenic shock	23/330 (6.97%)	24/149 (16.11%)	**0**.**002**
Cardiac arrest	49/331 (14.8%)	33/149 (22.15%)	**0**.**048**
Out-of-hospital CA	32/331 (9.67%)	17/149 (11.41%)	0.56
OTDT	3.0 (1.5–12.0)	5.0 (2.0–24.0)	**0**.**011**
Time from admission to wire crossing	92.0 (65.0–187.0)	115.0 (73.0–233.0)	**0**.**025**
AMI in history	61/320 (19.06)	10/147 (6.80)	**<0**.**001**
LVEF [%]	45.0 (38.0–50.0)	45.0 (35.0–50.0)	0.131
CCB	40/265 (15.09)	32/137 (23.36)	**0**.**041**
ARB	27/265 (10.19)	24/137 (17.52)	**0**.**036**
CCS			
1	13/290 (4.48)	17/119 (14.59)	**<0**.**001**
2	2/290 (0.69)	4/119 (3.36)	
3	12/290 (4.14)	19/119 (15.97)	
4	263/290 (90.69)	79/119 (66.39)	
NYHA			
1	244/290 (84.14)	66/100 (66)	**<0**.**001**
2	8/290 (2.76)	5/100 (5)	
3	8/290 (2.76)	16/100 (16)	
4	30/290 (10.34)	13/100 (13)	
Killip			
1	259/326 (79.45)	95/149 (63.76)	**<0**.**001**
2	26/326 (7.98)	29/149 (19.46)	
3	6/326 (1.84)	1/149 (0.67)	
4	35/326 (10.74)	24/149 (16.11)	
Grace	118.0 (100.0–146.0)	127.0 (108.0–153.0)	**0**.**008**
Euro score 2	4.08 (2.99–7.18)	2.64 (1.75–4.30)	**<0**.**001**
In-hospital mortality	26/331 (7.85)	21/149 (14.09)	**0**.**033**

Abbreviations: AMI, acute myocardial infarction; ARB, angiotensin II receptor blockers; BMI, body mass index; CA, cardiogenic shock; CCB, calcium channel blockers; CCS, Canadian Cardiovascular Society; CS, cardiogenic shock; LVEF, left ventricle ejection fraction; NYHA, New York Heart Association; OTDT, onset-to-door time.

Bolded values present statistically significant results of the analysis.

^a^ Data are presented as mean ± standard deviation, median (interquartile range), or counts (percentages).

### 3.5 Decline in the number of hospitalisations

In our study, 331 patients were enrolled in the pre-pandemic group during the 2-year observation time, and 149 patients were included in the pandemic group during 1-year of observation. Our analysis revealed that the number of STEMI cases admitted to the UCC per month deteriorated from 13.79 in the pre-pandemic group to 12.42 in the pandemic group. Furthermore, the number of patients admitted to the UCC with the diagnosis of STEMI per year declined by approximately 10% (165.5 vs. 149).

## 4 Discussion

In our report, we revealed the negative impact of the COVID-19 pandemic on STEMI treatment and results based on the time indicators and in-hospital mortality. For the first time, we are presenting prolongations in both time indicators—OTDT and time from admission to wire crossing- as well as an increase in in-hospital mortality and a decline in admission number because of STEMI diagnosis during the pandemic. Our results can be extrapolated to countries in comparable epidemic situation in this part of Europe.

OTDT is a combination of delays from both patients and EMS, however, due to the lack of specific data from EMS, we could not estimate these delays separately. Despite this limitation, indirect measurement can still provide valuable information and is widely used by researchers. In the Primessnig et al. study conducted in Berlin, Germany, pandemic group included 51 cases diagnosed with AMI. In the analysis of OTDT for STEMI cases, 23% of pandemic patients presented within the first 12 h from symptom onset, compared to 43% directly before the COVID-19 pandemic (*p* = 0.04). Similarly, 27% of cases presented after 72 h or more, compared to just 6% (8). Aldujeli et al. in a study conducted in Lithuania, in the pandemic group of 47 STEMI cases revealed a significant prolongation of median pain-to-door time compared to patients from the year prior (620 vs. 349 min; *p* = 0.0141). Data showed that 47% of patients in pandemic group presented more than 12 h after symptom onset, compared to 24% (*p* = 0.0127) (9). Significant prolongation in OTDT between the pandemic and the pre-pandemic group was also reported in a study conducted in Tokyo, Japan (241 min vs. 128 min, *p* = 0.028). Late presentations occurred in 26.5% vs. 12.1% cases, however, it was defined as OTDT >24 h compared to >12 h in European studies (10). Our study has confirmed recently reported diversities in OTDT. With a similar definition as in recent European studies, we reported 35.71% and 24.49% (*p* = 0.021) late presentations in pandemic and pre-pandemic groups, respectively. The possible reason are: avoidance of medical institutions due to patients' fear of SARS-Cov2 infection, imprecise modifications to the healthcare system for some patients which cause confusion in finding adequate clinic and difficulties to deliver EMS on time due to increased number of calls, as Jensen et al. study reported a 23.3% rise in EMS calls between 2019 and 2020 in Copenhagen, Denmark (11).

Although the ESC allowed for an additional 60 min due to the COVID-19 pandemic (6), we defined prolongation of time from patient admission to wire crossing based on recommendations (2). Several authors have reported changes in treatment times in their studies. Watanabe et al. in a study conducted in Tokyo, Japan observed an increase in delays between patients admitted before and during the pandemic (60 min vs. 72 min, *p* < 0.001), with a decline in number of individuals treated in recommended time frames from 74.8% to 63.6%, however, their analysis was limited to patients with a symptom onset-to-admission time ≤24 h (10). Aldujeli et al. study from Lithuania found a difference in the time from admission to PCI-mediated reperfusion that did not reach statistical significance (76 min vs. 86 min, *p* = 0.983) (9), although, the small study population is a serious limitation in this study. These results are consistent with Soylu et al. study involving 165 patients from both groups admitted to three PCI centres in Turkey that revealed a numerical disparity in systemic delay between the groups (69 min vs. 83 min, *p* = 0.076) (12). In a multi-national, retrospective registry study across 77 centres from 18 European countries authors enrolled 6,609 cases with the diagnosis of STEMI who underwent pPCI and reported significant prolongation in the time from admission to wire crossing after adjustment for population disparities (34 min vs. 36 min, *p*-0.007) (5). Our study with 480 enrolled patients, included more participants than any single-centre study and revealed an increase in the time from admission to PCI-mediated reperfusion between the pre-pandemic group and pandemic group that reached statistical significance (*p* = 0.025), aligning with the conclusions of De Luca et al. study. We believe it is due to necessity to wear PPE, delays in receiving SARS-CoV-2 antigen test results and transportation issues between the EU and the catheterization laboratory. Potential reasons for discrepancies between studies include small sample sizes, varying social restrictions, and differences in media communication methods between countries.

Management delays are among the most critical factors influencing mortality, alongside advanced age, Killip class, treatment strategy, and LVEF at admission (2). In the study utilizing data from 12 European countries with established national STEMI registries, including Poland, in-hospital mortality among non-selected patients varied widely, from 3% in Poland to 12% in Bulgaria, and from 2.2% in Macedonia to 6.1% in Bulgaria for patients treated with pPCI (13). In De Luca et al. study, in-hospital mortality increased from 4.9% to 6.8% (*p* < 0.001), comparing patients from March to April 2019 and 2020 from 77 European centres (5). Xiang et al. enrolled 25150 STEMI individuals from centres from the area of China in the early period of the pandemic. Patients were dichotomised to the Hubei sample, where the epidemiological condition was more severe, and to the non-Hubei sample. Authors reported an increase in in-hospital mortality compared to pre-pandemic groups (4.6% vs. 7.3%; *p* = 0.137 in the Hubei sample and 4.0% vs. 4.7%; *p* = 0.015 in the non-Hubei sample) (14). The number of cases in the Hubei group was approximately 30 times lower than in the non-Hubei group, which can affect the results of this study. Our analysis conducted on the group of 480 cases revealed the increase in in-hospital mortality when comparing the pre-pandemic and pandemic groups (7.85% vs. 14.09%, *p* = 0.033). The possible reasons for that rise are the prolongations of OTDT and time from admission to wire crossing and the poorer condition of patients at the admission, which is confirmed by the increased number of patients admitted with cardiogenic shock and cardiac arrest. Another reported observation is decrease in the number of AMI admissions. De Rosa et al. conducted a multi-centre, nationwide, observational study in Italy in the early pandemic which revealed a 48.4% decline in patients referred to cardiac care units. Although the most relevant alterations concerned NSTEMI, where the number of cases decreased by 65.1%, the reduction in STEMI patients in this study reached 26.5% (4). Comparable findings were reported by De Luca et al. in the analysis performed in 77 European PCI centres, where deterioration in STEMI cases reached 18.9% (5). Xiang et al. study reported reduction in hospitalisation due to STEMI diagnosis reached 26% in the non-Hubei area and approximately 62% in the Hubei group, indicating the severity of the COVID-19 pandemic as the potential source (14). Meta-analysis of 10 studies conducted by Rattka et al. confirm the reduction in the number of STEMI cases (15). Our analysis revealed an approximately 10% decline in patients with STEMI diagnoses admitted to the UCC annually—the monthly number of patients reduced from 13.79 to 12.42.

Modified healthcare organisation with several centres dedicated to COVID-19-positive patients exclusively restrained the access for patients with other urgent medical states, including STEMI. There are numerous reasons for the disparities in the results between studies. Countries were affected by the COVID-19 pandemic in different severities, and diverse resources were used to confront the situation. The disparities between governments' limitations and modifications in local STEMI treatment guidelines also affect the findings. Preceding observations make it challenging to compare the results of studies between countries.

## 5 Study limitations

Several limitations that occurred in the study have to be mentioned. First, our study is retrospective, observational and single-centre, making the results not fully applicable to other regions. Secondly, our study population is relatively homogenous, including patients from the agglomeration of Tricity and surrounding localities. Furthermore, data was collected during the pandemic, increasing the probability of aberrations in the reports. Nevertheless, we made every effort to provide the most reliable data. Finally, in our study, we enrolled patients from the early period of the pandemic; subsequently, our observations may overestimate the influence of the COVID-19 pandemic on STEMI management.

## 6 Conclusions

In conclusion, our study demonstrated the negative impact of the COVID-19 pandemic on the healthcare system in Poland by evaluating treatment times and outcomes in STEMI patients. We found significant prolongations in both estimated treatment times for patients admitted during the pandemic. This can likely be attributed to an increased number of EMS activations and additional epidemic protocols, such as PCR testing at admission and the requirement for medical professionals to wear PPE. Furthermore, the treatment outcome indicator was also affected, with in-hospital mortality significantly higher in the pandemic group. Patients admitted to our tertiary multidisciplinary teaching center during the pandemic had a greater risk of cardiac arrest or cardiogenic shock, indicating a poorer condition at admission compared to the pre-pandemic period. This observation suggests a potential link between increased in-hospital mortality and prolonged OTDT and time from admission to wire crossing. Finally, the number of patients admitted to our centre declined, possibly due to patients' fear of infection during hospitalization or lack of information about modified healthcare organization.

## Data Availability

The data analyzed in this study is subject to the following licenses/restrictions: authors will share the dataset on reasonable request. Requests to access these datasets should be directed to Jakub Bychowski, jakub.bychowski@gumed.edu.pl.
